# Supplementary figures and images for: Tumor-Suppressive Effect of Metformin via the Regulation of M2 Macrophages and Myeloid-Derived Suppressor Cells in the Tumor Microenvironment of Colorectal Cancer
Source: Cancers (Basel). 2022 Jun 10;14(12):2881. doi: 10.3390/cancers14122881 (PMC9220791; doi:10.3390/cancers14122881)

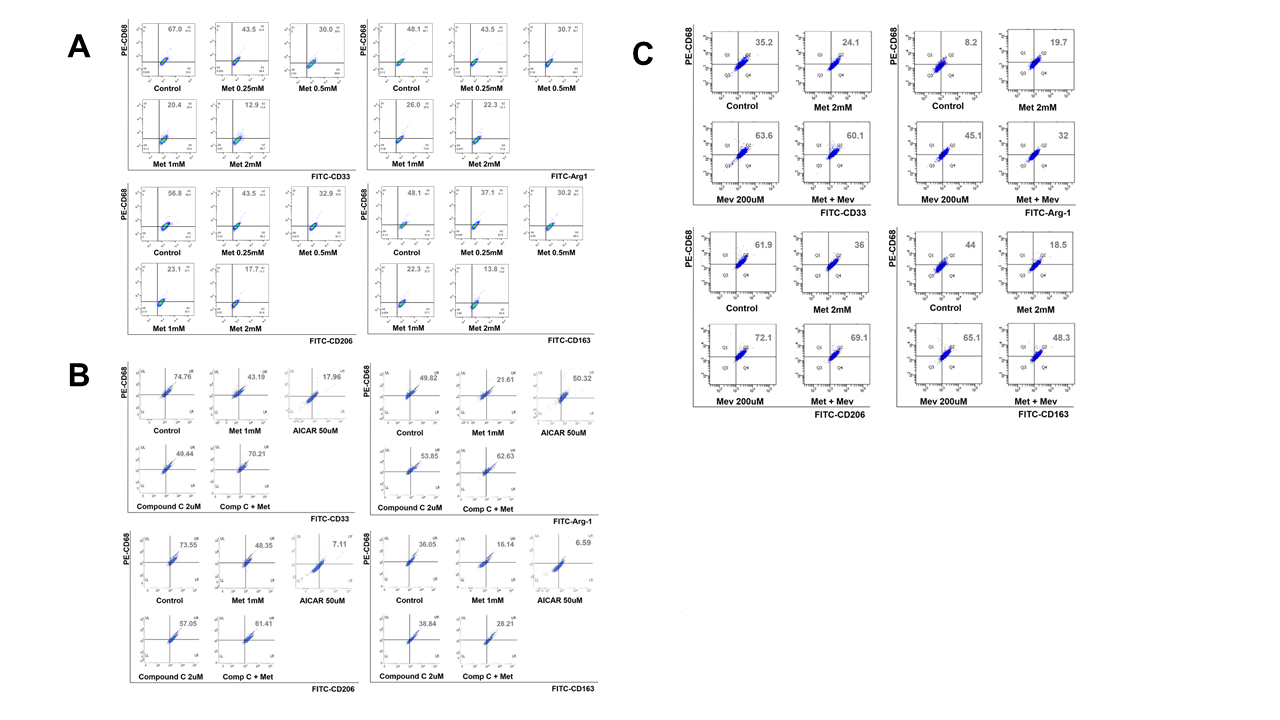

Supplement: Supplementary file 1 [file cancers-14-02881-s001.zip › Figure S1.tif]

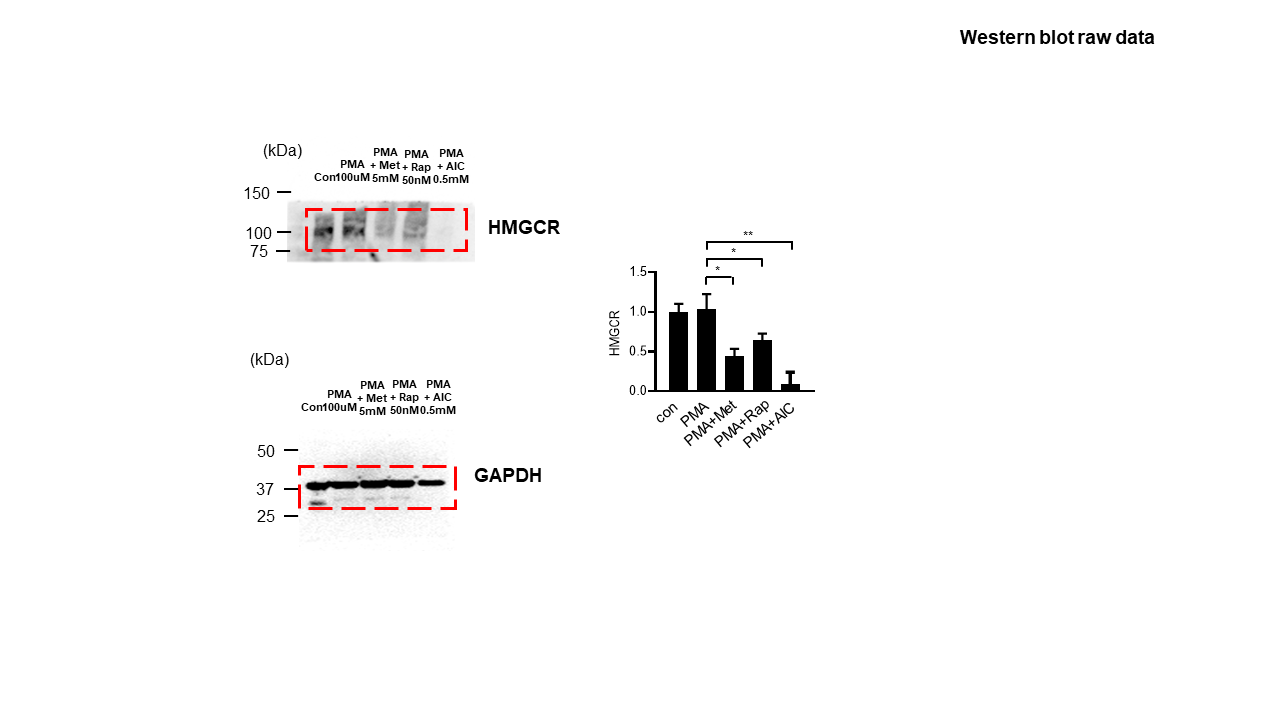

Supplement: Supplementary file 1 [file cancers-14-02881-s001.zip › Figure S10.tif]

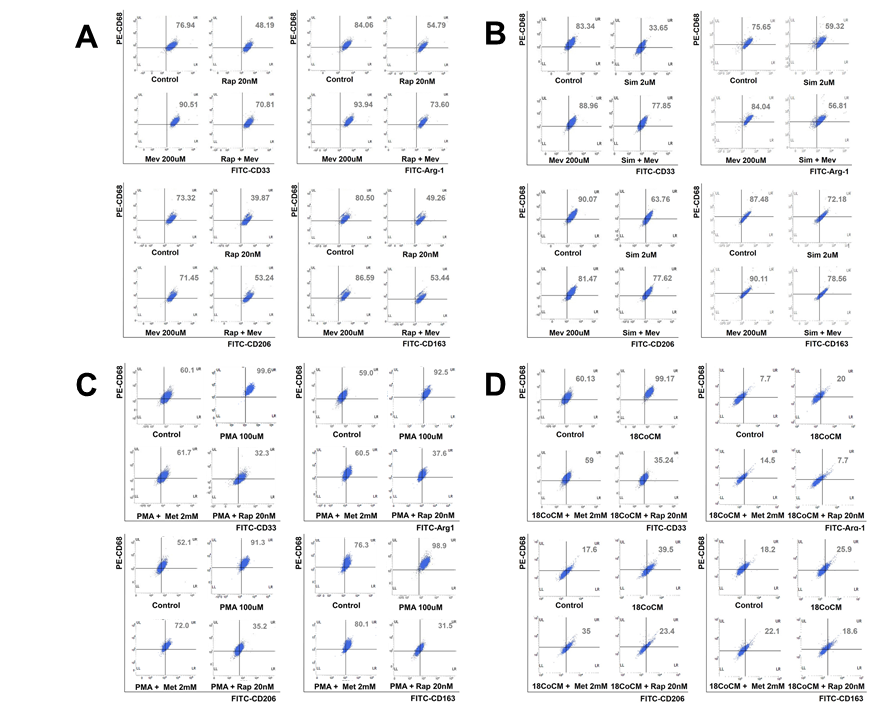

Supplement: Supplementary file 1 [file cancers-14-02881-s001.zip › Figure S2.tif]

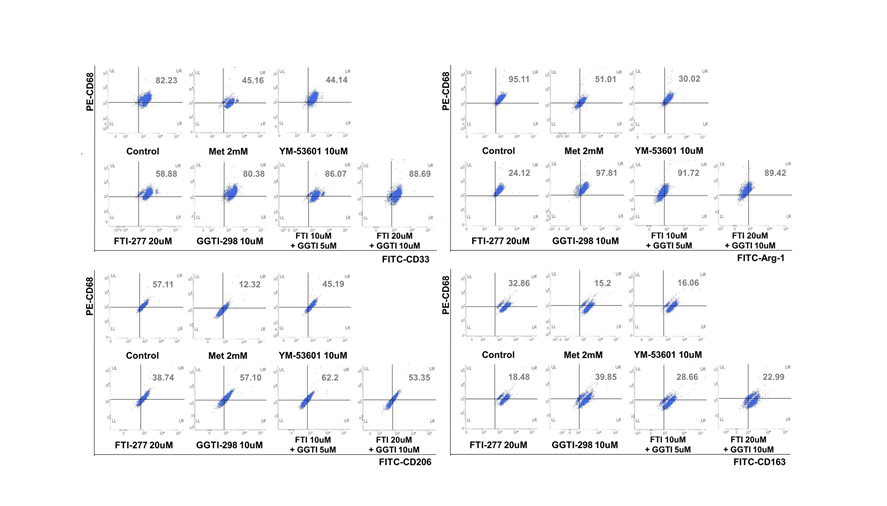

Supplement: Supplementary file 1 [file cancers-14-02881-s001.zip › Figure S3.tif]

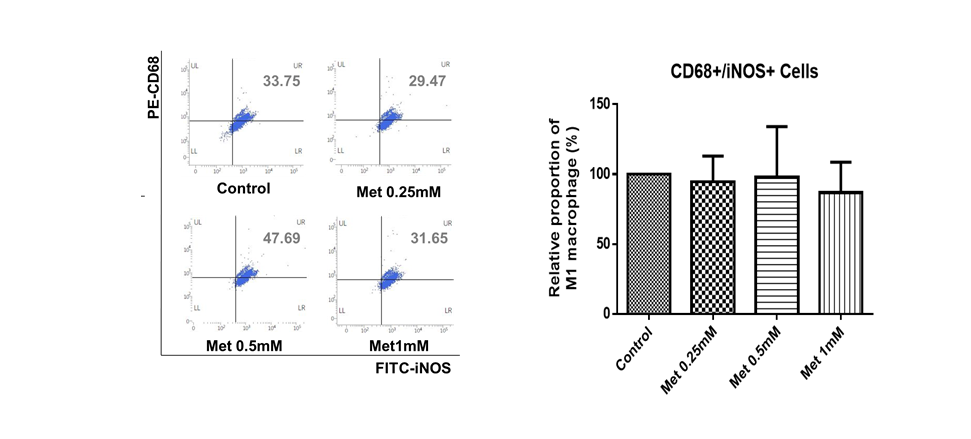

Supplement: Supplementary file 1 [file cancers-14-02881-s001.zip › Figure S4.tif]

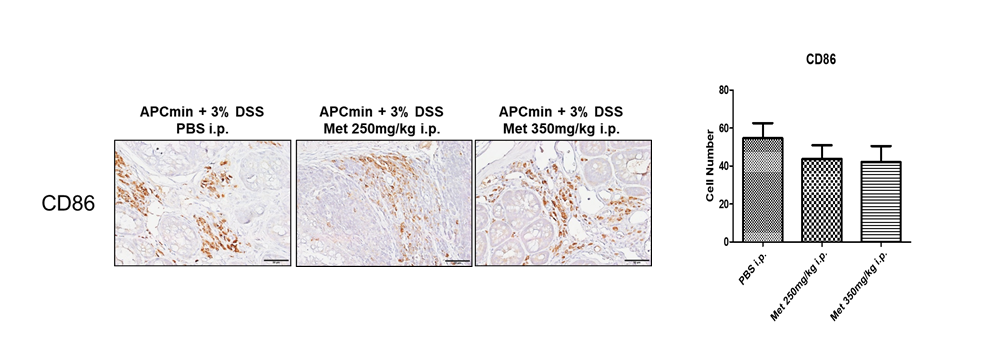

Supplement: Supplementary file 1 [file cancers-14-02881-s001.zip › Figure S5.tif]

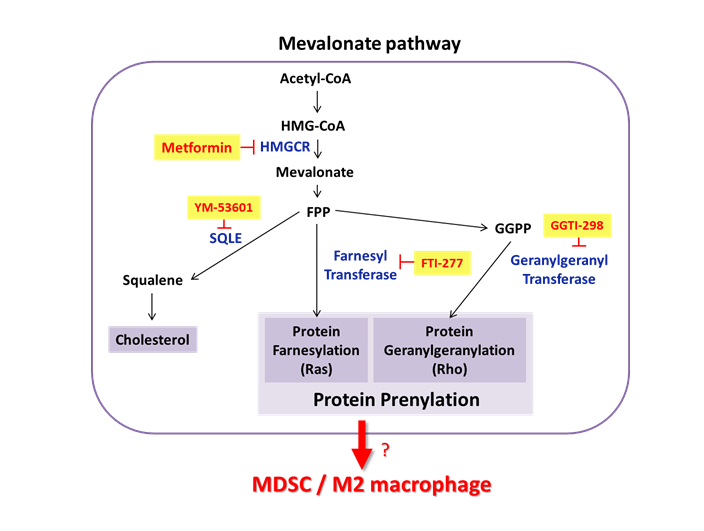

Supplement: Supplementary file 1 [file cancers-14-02881-s001.zip › Figure S6.tif]

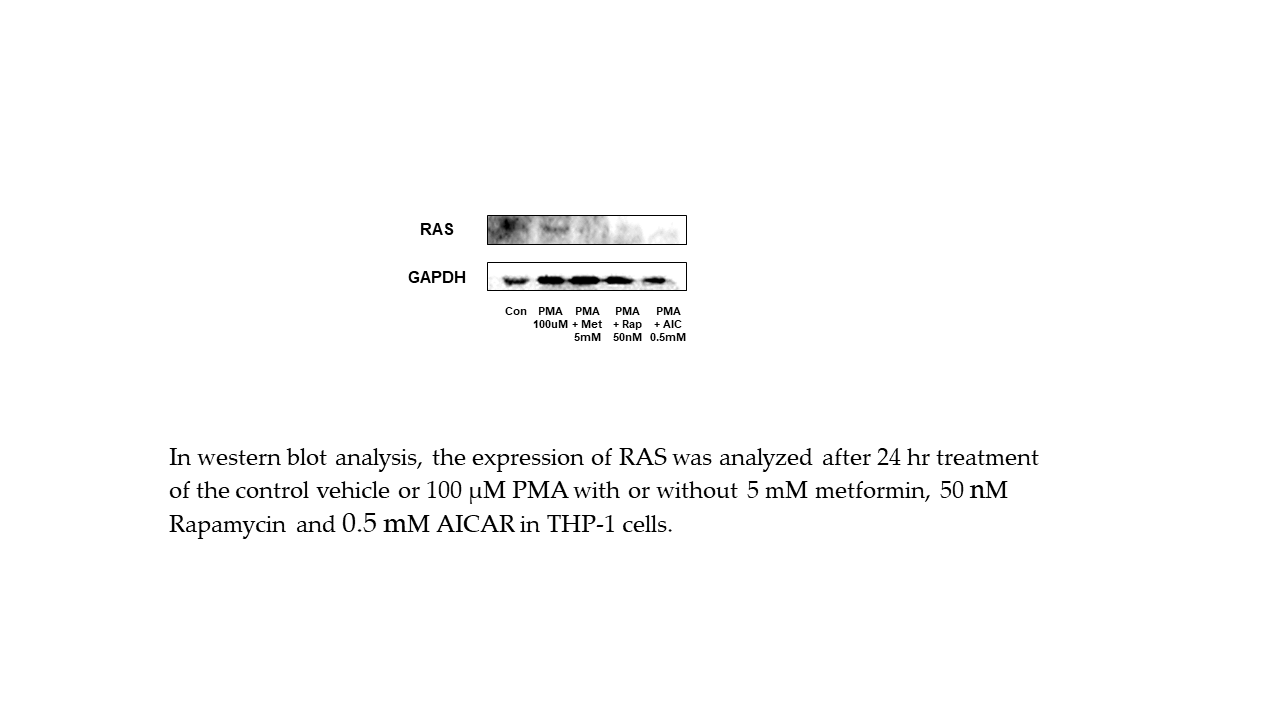

Supplement: Supplementary file 1 [file cancers-14-02881-s001.zip › Figure S7.tif]

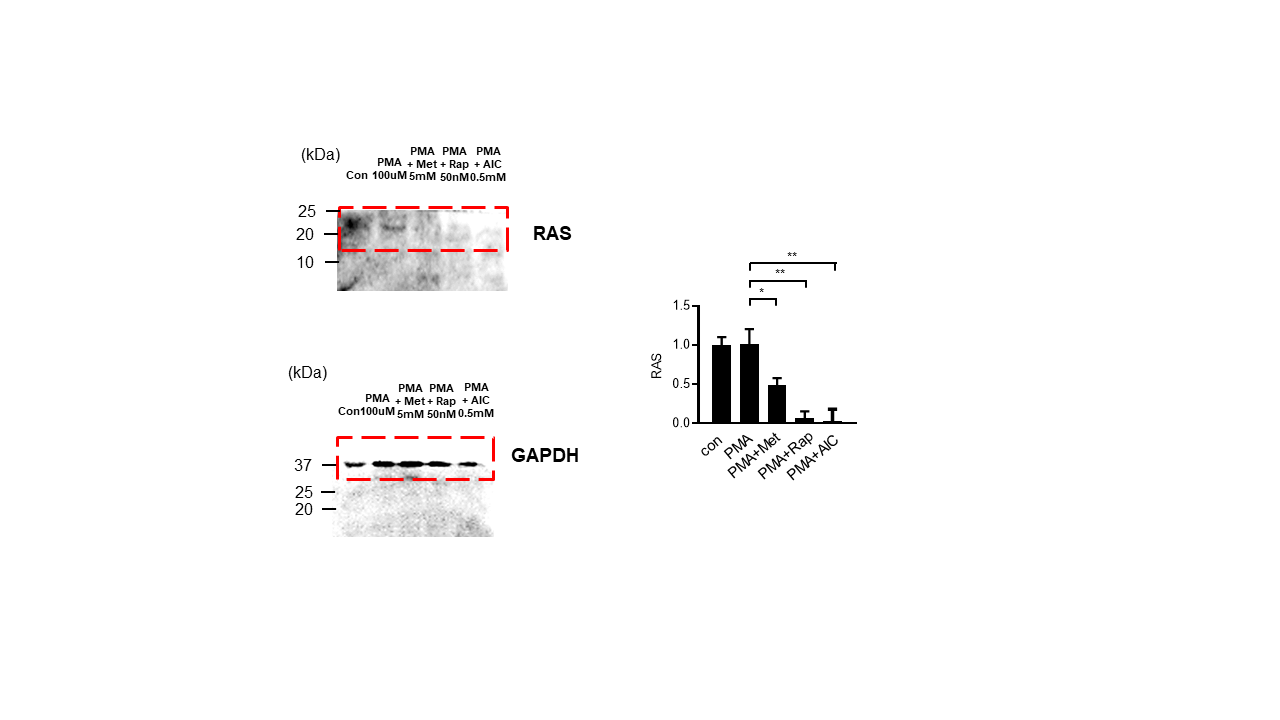

Supplement: Supplementary file 1 [file cancers-14-02881-s001.zip › Figure S8.tif]

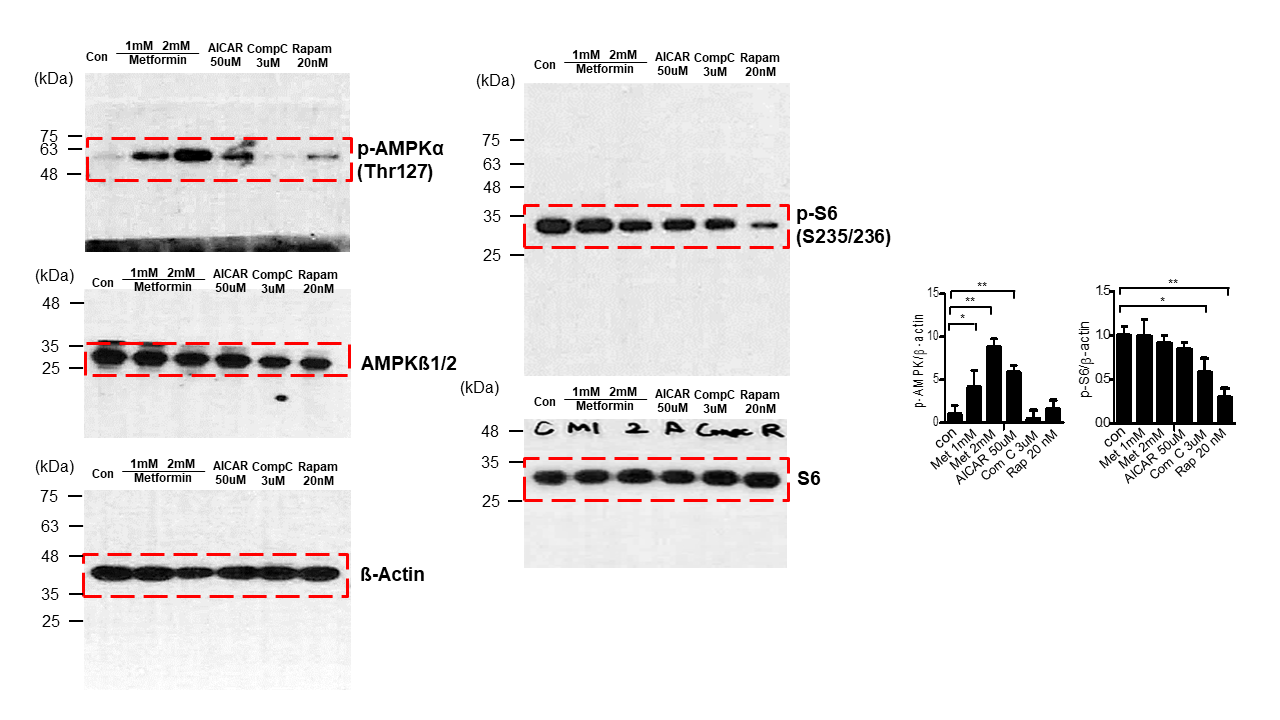

Supplement: Supplementary file 1 [file cancers-14-02881-s001.zip › Figure S9.tif]
